# Hormonal contraceptives and risk of ischemic stroke in women with migraine: a consensus statement from the European Headache Federation (EHF) and the European Society of Contraception and Reproductive Health (ESC)

**DOI:** 10.1186/s10194-017-0815-1

**Published:** 2017-10-30

**Authors:** Simona Sacco, Gabriele S. Merki-Feld, Karen Lehrmann Ægidius, Johannes Bitzer, Marianne Canonico, Tobias Kurth, Christian Lampl, Øjvind Lidegaard, E. Anne MacGregor, Antoinette MaassenVanDenBrink, Dimos-Dimitrios Mitsikostas, Rossella Elena Nappi, George Ntaios, Per Morten Sandset, Paolo Martelletti, on behalf of the European Headache Federation (EHF) and the European Society of Contraception and Reproductive Health (ESC)

**Affiliations:** 1 Department of Applied Clinical Sciences and Biotechnology, University of L’Aquila, L’Aquila, Italy; 2 Department of Gynecology, Clinic for Reproductive Endocrinology, University Hospital, Zürich, Switzerland; 3 Department of Neurology, Bispebjerg Hospital and University of Copenhagen, Copenhagen, Denmark; 4 Department of Obstetrics and Gynecology, University Hospital of Basel, Basel, Switzerland; 5 Université Paris-Saclay, University Paris-Sud, UVSQ, CESP, Inserm UMRS1018, Orsay, France; 6 Institute of Public Health, Charité – Universitätsmedizin Berlin, Berlin, Germany; 7 Headache Medical Center Seilerstaette Linz, Linz, Austria; 8 Department of Geriatric Medicine Ordensklinikum Linz, Linz, Austria; 9 Department of Obstetrics & Gynaecology, Rigshospitalet, Faculty of Health Sciences, University of Copenhagen, Copenhagen, Denmark; 10 Centre for Neuroscience & Trauma, BICMS, Barts and the London School of Medicine and Dentistry, London, UK; 11 Barts Sexual Health Centre, St Bartholomew’s Hospital, London, UK; 12 Division of Vascular Medicine and Pharmacology, Department of Internal Medicine, Erasmus Medical Center Rotterdam, Rotterdam, The Netherlands; 13 Department of Neurology, Aeginition Hospital, National and Kapodistrian University of Athens, Athens, Greece; 14 Research Centre for Reproductive Medicine, Gynecological Endocrinology and Menopause, IRCCS S. Matteo Foundation, Department of Clinical, Surgical, Diagnostic and Pediatric Sciences, University of Pavia, Pavia, Italy; 15 University Consortium for Adaptive Disorders and Head Pain (UCADH), University of Pavia, Pavia, Italy; 16 Department of Medicine, University of Thessaly, Larissa, Greece; 17 Department of Haematology, Oslo University Hospital and University of Oslo, Oslo, Norway; 18 Department of Clinical and Molecular Medicine, Sapienza University of Rome, Rome, Italy; 19 Regional Referral Headache Centre, Sant’Andrea Hospital, Rome, Italy

**Keywords:** Migraine, Aura, Headache, Stroke, Hormonal contraceptives, Contraception

## Abstract

Several data indicate that migraine, especially migraine with aura, is associated with an increased risk of ischemic stroke and other vascular events. Of concern is whether the risk of ischemic stroke in migraineurs is magnified by the use of hormonal contraceptives. As migraine prevalence is high in women of reproductive age, it is common to face the issue of migraine and hormonal contraceptive use in clinical practice. In this document, we systematically reviewed data about the association between migraine, ischemic stroke and hormonal contraceptive use. Thereafter a consensus procedure among international experts was done to develop statements to support clinical decision making, in terms of cardiovascular safety, for prescription of hormonal contraceptives to women with migraine. Overall, quality of current evidence regarding the risk of ischemic stroke in migraineurs associated with the use of hormonal contraceptives is low. Available data suggest that combined hormonal contraceptive may further increase the risk of ischemic stroke in those who have migraine, specifically migraine with aura. Thus, our current statements privilege safety and provide several suggestions to try to avoid possible risks. As the quality of available data is poor further research is needed on this topic to increase safe use of hormonal contraceptives in women with migraine.

Several case-control and cohort studies, as well as pooled data analyses, indicate that migraine is a risk factor for stroke and other vascular events [1–6]. Most of the evidence supports an increased risk of ischemic stroke associated with migraine with aura [7–18]. For migraine without aura, the interpretation of available data is more complex as some studies reported that migraine without aura is also associated with an increased risk of ischemic stroke [10, 18, 19] whereas in others the association was not confirmed [7, 11, 15, 16]. Further, the definition of a clear association between migraine without aura and ischemic stroke is even more complex as some studies did not include information on migraine aura [2, 18, 20, 21] and because of the challenge of aura diagnosis in epidemiological studies. Two meta-analyses did not demonstrate an association between migraine without aura and ischemic stroke [5, 6]. Whether the risk of ischemic stroke in women with migraine is magnified by use of combined hormonal contraceptives (HCs) is unclear. As migraine prevalence is high in women of reproductive age it is common to face the issue of migraine and HC use in clinical practice [22]. Hence, the aim of the present paper is to systematically review the association between migraine, ischemic stroke and HC use and to develop a consensus among international experts to support clinical decision-making in terms of cardiovascular safety for prescription by healthcare professionals of HCs to women with migraine.

## Methods

In July 2016, European Headache Federation (EHF) representatives selected a panel of international multidisciplinary experts in migraine, cardiovascular risks and hormonal contraception. The panel was chosen to represent the breadth of knowledge and experience and a wide variety of opinions internationally. The focus of this statement is to provide guidance for clinicians. From the original selected panel, two out of 17 members who initially agreed to participate decided to leave the group, and they are not included among the Panel members.

### Review of the literature

A systematic search of the literature was conducted to identify key papers addressing the association between migraine and cardiovascular events in women using HCs. An initial literature search included all papers indexed on PubMed and Scopus, from inception to August 30, 2016. The following search terms were used in both databases: “migraine” AND (contraceptive OR estrogen) AND (vascular OR stroke OR “myocardial infarction” OR angina OR “coronary artery disease” OR “coronary heart disease” OR “venous thrombosis”). Both observational (cross-sectional, case-control, and cohort) and intervention studies (RCTs) were included.

Two investigators from the statement Supporting Group (FP and RO) independently screened the titles and abstracts of the publications identified to verify study eligibility. Full texts of selected studies were evaluated when appropriate. Disagreements were resolved by consensus. The reference lists and Google Scholar citations of the selected articles were also screened. To summarize the search results, a data extraction sheet was developed including the information of interest. A formal systematic review of the association between migraine and cardiovascular events and HCs as well as the risk of vascular events was not performed and is available elsewhere [3–6, 23–26].

Papers retrieved from the literature search as well as summary Tables were shared among the panelists before starting the consensus procedure. The systematic literature search was repeated at the end of the consensus procedure to include all relevant papers published until March 30, 2017.

### Development of the expert consensus

The consensus process incorporates a modified Delphi method [27]. The Delphi method is largely used in the healthcare setting as a reliable means of determining consensus for a defined clinical problem [28–30]. This method is an iterative process that uses a systematic progression of repeated rounds of voting and is an effective process for determining expert group consensus where there is little or no definitive evidence and where opinion is important.

Development of the consensus statement was organized in four rounds. In each round, panelists were instructed not to discuss among themselves and to send their feedbacks only to the facilitator (SS). Two core panelists (SS, PM) developed a draft document containing the list of items to be included in the statements based on available literature and on clinical grounds. The items were constructed as open-ended questions and the document was used for soliciting information from the panelists. In round 1, the draft containing the questions was sent by e-mail to all panelists accompanied by a clear explanation of the objectives of the study and specific instructions. Panelists were asked to provide free-text responses for each of the open questions and to suggest additional items of relevance as warranted. Thereafter, the facilitator analysed answers obtained during round 1 and drafted the statements. In round 2, the draft of the statements was sent by e-mail to all panelists. Each panelist was asked to rate their agreement for each statement by marking “completely agree”, “partly agree (modifications required)” or “disagree” beside each statement. Where panelists selected “partly agree” or “disagree” for a statement, they were asked to provide a free-text explanation for their selection. Panelists were also given the opportunity to provide comments and suggestions and to identify further additional items not included in the initial list of statements. Responses were then analysed by the facilitator and used to refine statements. In round 3, a revised draft was developed and emailed to all panelists and the panelists were asked again to vote their agreement, as in round 2, but with the knowledge of the group scores and comments. Thus, participants could reflect upon the group results and change their mind, while preserving the anonymity of their responses. Final responses were then analysed and statements further refined. In round 4, a final draft of the statements was e-mailed to all panelists. Panelists were asked to simply express agreement or disagreement for each statement without further comments. The panelists were also required to provide a rank order of the statements. Response frequencies for each item were calculated and entered anonymously into a database. Statements to be included in the final document required 80% agreement from the panel [31].

### Drafting of the statements

Quality of evidence and strength of the recommendations were rated according to the American College of Chest Physicians (ACCP) Task Force (Table 1) [32]. We also used the suggestions provided by the ACCP referring to wording of the recommendations. When making a strong recommendation we used the terminology “We recommend…” whereas when making a weak recommendation, less definitive wording was used, such as, “We suggest…” was used.

**Table 1:** Grading recommendations according to the American College of Chest Physicians (ACCP) Task Force

Grade of Recommendation/Description	Benefit vs Risk and Burdens	Methodological Quality of Supporting Evidence	Implications
1A/strong recommendation, high-quality evidence	Benefits clearly outweigh risk and burdens, or vice versa	RCTs without important limitations or overwhelming evidence from observational studies	Strong recommendation, can apply to most patients in most circumstances without reservation
1B/strong recommendation, moderate quality evidence	Benefits clearly outweigh risk and burdens, or vice versa	RCTs with important limitations (inconsistent results, methodological flaws, indirect, or imprecise) or exceptionally strong evidence from observational studies	Strong recommendation, can apply to most patients in most circumstances without reservation
1C/strong recommendation, low-quality or very low quality evidence	Benefits clearly outweigh risk and burdens, or vice versa	Observational studies or case series	Strong recommendation but may change when higher quality evidence becomes available
2A/weak recommendation, high quality evidence	Benefits closely balanced with risks and burden	RCTs without important limitations or overwhelming evidence from observational studies	Weak recommendation, best action may differ depending on circumstances or patients’ or societal values
2B/weak recommendation, moderate-quality evidence	Benefits closely balanced with risks and burden	RCTs with important limitations (inconsistent results, methodological flaws, indirect, or imprecise) or exceptionally strong evidence from observational studies	Weak recommendation, best action may differ depending on circumstances or patients’ or societal values
2C/weak recommendation, low quality or very low-quality evidence	Uncertainty in the estimates of benefits, risks, and burden; benefits, risk, and burden may be closely balanced	Observational studies or case series	Very weak recommendations; other alternatives may be equally reasonable

From Baumann MH et al. Chest 2001;119:590–602; RCT indicates randomized controlled trial

## Results

### Systematic review

**Fig. 1:**
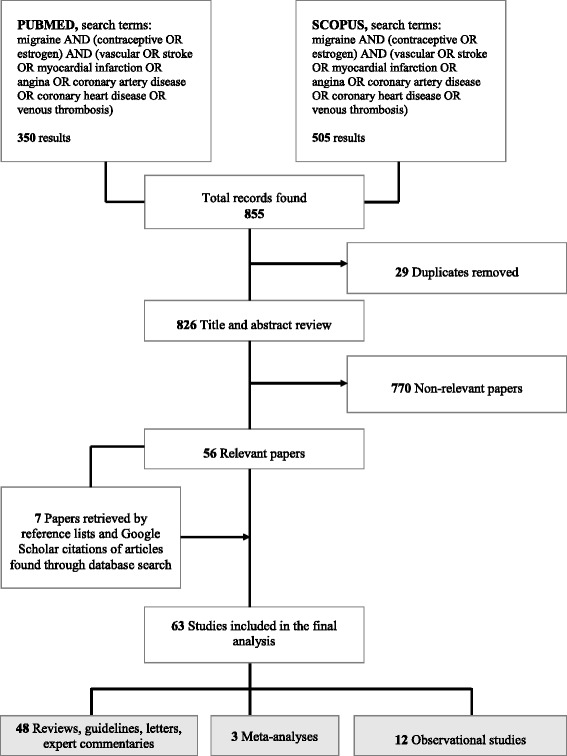
Flow-chart for the systematic review

The systematic review of the literature identified six studies that evaluated if HC use increased the risk of stroke (ischemic, hemorrhagic, or both) in women with migraine [10, 11, 17, 18, 33, 34]: four studies that evaluated if the association between migraine and cardiovascular diseases was modified by HC use [2, 35–37], and two studies that reported descriptive information about HC use and occurrence of stroke in women with migraine [38, 39] Fig. 1.

Characteristics of the original research studies included in the systematic review are reported in Table 2. All the studies reporting stroke risk in women with migraine according to HC use had a cross-sectional design and were case-control and hospital-based [11, 17, 18, 33, 34] with the exception of one population-based study [10]; two of the studies reporting interaction by HC use had a prospective or retrospective cohort design [2, 35] while other two studies had a case-control design [36, 37]; the two studies reporting descriptive statistics had a cross-sectional hospital-based design [38, 39]. HCs used in the selected studies included pills containing ≥50 μg estrogen, 30–40 μg estrogen, 20 μg estrogen, or progestogen only; seven studies did not specify the type of HC [2, 10, 17, 35, 37–39].

**Table 2:** Characteristics of the original research studies included in the systematic review

Study	Population	Design	Sample size	Study period	Outcome of interest	Exposure	Diagnostic criteria for migraine	Confirmation of stroke diagnosis	Type of hormonal contraceptive	Quality of evidence (ACCP)
Collaborative Group, 1975 [33]	Women with stroke aged 15–44 years	Hospital-based, case-control	430 strokes, 429 hospital controls, 451 neighborhood controls	1969–71	Ischemic stroke	Migraine vs no migraine	Interview; no ICHD	Yes	Estrogen ≥50 μg	C
Tzourio, 1995 [18]	Women with stroke aged <45 years	Hospital-based, case-control	72 strokes, 173 controls	1990–93	Ischemic stroke	Migraine vs no migraine	Questionnaire; ICHD	Yes	Estrogen 50 μg (14.6%), 30–40 μg (73.8%), 20 μg (6.8%), progestogen only (4.8%)	C
Lidegaard, 1995 [36]	Women aged 15–44 years	Hospital-based, case-control	497 ischemic strokes, 1396 controls	1985–1989	Ischemic stroke	Migraine vs no migraine, HC use vs non-use	Questionnaire; no ICHD	No	Estrogen 50 μg (4.5%), 30–40 μg (11.0%), progestogen only (1.5%), unspecified (1.6%)	C
Schwartz, 1998 [34]	Women with stroke aged 18–44 years	Hospital-based, case-control	373 strokes, 1191 controls	1991–95	Ischemic stroke	Migraine vs no migraine	Interview; no ICHD	Yes	Estrogen <50 μg	C
Chang, 1999 [11]	Women with stroke aged 20–44 years	Hospital-based, case-control	291 strokes, 736 controls	1990–94	Ischemic stroke	Migraine vs no migraine	Questionnaire; ICHD	Yes	Estrogen ≥50 μg and <50 μg (stratified data)	C
Milhaud, 2001 [38]	Women with ischemic stroke	Hospital-based, cohort	3502 ischemic strokes (130 migraineurs)	1979–98	NA	NA	Interview; ICHD	Yes	Not specified	C
Nightingale, 2004 [37]	Women aged 15–49 years	Hospital-based, case-control	190 strokes, 1129 controls	1992–1998	Ischemic stroke	Migraine vs no migraine, HC use vs non-use	Administrative code (GPRD); no ICHD	Yes	Not specified	C
MacClellan, 2007 [17]	Women with ischemic stroke aged 15–49 years	Hospital-based, case-control	386 ischemic strokes, 614 controls	2001–03	Ischemic stroke	Migraine with aura vs no migraine	Questionnaire; ICHD	Yes	Not specified	C
Pezzini, 2011 [39]	Subjects with ischemic stroke aged ≤45 years	Hospital-based, cohort	981 strokes (235 migraineurs, 50.6% women)	2000–09	NA	NA	Interview; ICHD	Yes	Not specified	C
Kurth, 2016 [2]	Women aged 25–42 years at baseline	Prospective, cohort	115,541 women	1989–2011	Stroke	Migraine with and without aura vs no migraine	Questionnaire; ICHD	No	Not specified	B
Albieri, 2016 [35]	Subjects aged 25–80 years (subanalysis in women aged 25–50 years)	Retrospective, cohort	49,711 ischemic strokes	2003–2011	Stroke	Triptan prescription	Administrative code (triptan prescription); No ICHD	No	Not specified	C
Champaloux, 2017 [10]	Women aged 15–49 years	Population-based, case-control	25,887 ischemic strokes	2006–2012	Ischemic stroke	Migraine with or without aura vs no migraine	Administrative code (ICD-9-CM); Nno ICHD		Not specified	C

NA indicates not applicable; GPRD indicates General Practice Research Database; HC indicates hormonal contraceptives; ICD-9-CM indicates International Classification of Diseases, Ninth Revision, Clinical Modification; ICHD indicates International Classification of Headache Disorders

The results of studies addressing the relationship among migraine, HC use, and ischemic stroke are summarized in Table 3. A case-control study showed that the risk of ischemic stroke was increased by 2-fold in migraineurs who were HC users (odds ratio [OR] 2.1; 95% CI 1.2–3.7) as compared to women with migraine who were HC non-users [34], while another case-control study found a 7-fold higher risk of ischemic stroke (OR 7.0; 95% CI 1.4–22.8) in smokers and HC users compared with non-smokers and non-HC users among women with migraine with aura [17]. An early case-control study comparing women with stroke versus stroke-free female control subjects showed that the risk was increased by 5- to 6-fold in women with migraine who were HC users (relative risk [RR] 4.6; 95% confidence interval [CI] 2.2–9.6 for hospital controls and 5.9; 2.9–12.2 for neighbor controls) and by 2-fold in women with migraine who were HC non-users (RR 2.0; 95% 1.2–3.3; comparison to neighbor controls) as compared to women without migraine [33]. A subsequent case-control study showed that the risk was increased by 14-fold in women with migraine who were HC users (odds ratio [OR] 13.9; 95% CI 5.5–35.1) and by 4-fold in women with migraine who were HC non-users (OR 3.7; 95% CI 1.5–9.1) as compared to women without migraine who were HC non-users [18]. A further case-control study showed that the risk was increased by 17-fold in women with migraine who were HC users (OR 16.9; 95% CI 2.7–106) but was unable to demonstrate an association in women with migraine who were HC non-users as compared to women without migraine who were HC non-users [11]. Three case-control studies investigated the interaction among migraine, HC use, and smoking in the determination of the risk of ischemic stroke [11, 17, 36]; the first study found an adjusted OR for ischemic stroke in migraineurs of 2.8 (*P* < 0.01; CI not reported), in the absence of synergism between migraine, HC use, and smoking [36]; another study found an OR of 34.4 (95% CI 3.27–361) for ischemic stroke in women with migraine who used HC and smoked compared with women without migraine, non-HC users, and non-smokers [11]; the other study found an OR for ischemic stroke of 10.0 (95% CI 1.4–73.7) when comparing women with migraine with aura who used HC and smoked with women without migraine who were non-HC users and non-smokers [17]. Two studies were performed in young subjects with ischemic stroke [38, 39]; the first study found that HC use was similar in women with migraine and in the referent group (45% vs 30%; *P* = 0.056) and, in the logistic regression analysis, HC use was a risk factor for migraine [38]. The other study found that in a cohort of ischemic stroke patients, HC use was similar in women with migraine (with and without aura) and in the referent group (36.7% vs 33.3% vs 36.8%; *P* = 0.905) [39]. One population-based, case-control study reported separate results for women with migraine with and without aura, and for HC users and non-users; in that study, the combination of HC use and migraine with aura was associated with an OR of 6.1 (95% CI 3.1–12.1) for ischemic stroke in women aged 15–49 years and the combination of HC use and migraine without aura was associated with an OR of 1.8 (95% CI 1.1–2.9) in the same risk [10]. Three studies reported the interaction between migraine and HC use in the determination of the risk of ischemic stroke [2, 35, 37]; in the first study, an increased OR for ischemic stroke was found in subjects with history of migraine compared with women without migraine (2.3, 95% CI 1.0–5.2), and in current HC users compared with non-users (2.3, 95% CI 1.2–4.6), in the absence of a significant interaction between migraine and HC use [37]. Another study showed an increased risk of major cardiovascular events and of cardiovascular mortality in women with migraine as compared to women without that was not modified by HC use (P for interaction all ≥0.84) [2]. A further study assessed the risk of any stroke (both ischemic and hemorrhagic) among triptan users 25–50 years of age and found a hazard ratio (HR) of 1.4 (95% CI 1.3–1.7) which was not changed after oral contraceptives were also adjusted for [35]. However, whether the effect of triptan use on stroke outcomes was modified by oral contraceptive was not reported.

**Table 3:** Risk of ischemic stroke in subjects with migraine according to use of hormonal contraceptives

Study	Population	Comparison	Odds ratio (95% confidence interval)	Adjustment factors
Any migraine				
Collaborative Group, 1975 [33]	Women with stroke, hospital controls	Women with migraine and HC use vs non-migraineurs, non-HC users	4.6 (2.2–9.6)*	Age
	Women with stroke, neighbor controls	Women with migraine and HC use vs non-migraineurs, non-HC users	4.6 (2.2–9.6)*	Age
Lidegaard 1995 [36]	Women with ischemic stroke, controls	Smokers, HC users, and migraineurs vs non-smokers, non-HC users, and non-migraineurs	3.3 (P < 0.01)	HC, diabetes, arterial hypertension, other diseases
Tzourio, 1995 [18]	Women with stroke, controls	Women with migraine and HC use vs non-migraineurs, non-HC users	13.9 (5.5–35.1)	Not reported
Schwartz, 1998 [34]	Women with migraine	HC users vs non-HC users	2.1 (1.2–3.7)	Age, smoking, body mass index
Chang, 1999 [11]	Women with stroke, controls	Women with migraine and HC use vs non-migraineurs, non-HC users	16.9 (2.7–106)	Hypertension, education, smoking, family history of migraine, alcohol use, social class
	Women with stroke, controls	Women with migraine, smoke, and HC use vs non-migraineurs, non-smokers, and non-HC users	34.4 (3.3–361)	Not reported
Milhaud, 2001 [38]	Women with ischemic stroke	Women with migraine and HC use vs non-migraineurs, non-HC users	2.7 (1.2–6.0)	Not reported
Migraine with aura				
MacClellan, 2007 [17]	Women with migraine with aura	Smokers and HC users vs non-smokers and non-HC users	7.0 (1.4–22.8)	Age, race, geographic region, study period
	Women with stroke, controls	Women with migraine with aura, smokers, and HC users, vs non-migraineurs, non-smokers, and non-HC users	10.0 (1.4–73.7)	Age, race, geographic region, study period
Champaloux, 2017 [10]	Women with stroke, controls	Women with migraine with aura and HC users vs non-migraineurs and HC users	6.1 (3.1–12.1)	Hypertension, diabetes, obesity, smoking, ischemic heart disease, valvular heart disease
	Women with stroke, controls	Women with migraine with aura and non-HC users vs non-migraineurs and non-HC users	2.7 (1.9–3.7)	Hypertension, diabetes, obesity, smoking, ischemic heart disease, valvular heart disease
Migraine without aura				
Champaloux, 2017 [10]	Women with stroke, controls	Women with migraine without aura and HC users vs non-migraineurs and HC users	1.8 (1.1–2.9)	Hypertension, diabetes, obesity, smoking, ischemic heart disease, valvular heart disease
	Women with stroke, controls	Women with migraine without aura and non-HC users vs non-migraineurs and non-HC users	2.2 (1.9–2.7)	Hypertension, diabetes, obesity, smoking, ischemic heart disease, valvular heart disease

*Relative risk; HC indicates hormonal contraceptives

### Risk of ischemic stroke associated with the use of hormonal contraceptives

We calculated absolute risks of ischemic stroke in young women according to migraine status and hormonal contraceptive use (Table 4). Data were calculated considering an estimated incidence of ischemic stroke in women aged 25 to 44 years of 2.5/100,000 [37]. For migraine with aura we considered a pooled RR of ischemic stroke of 2.3 (95%CI 1.4–3.9, heterogeneity: Q = 8.3, df = 3, *p* = 0.039, I^2^ = 64%) using a random effect model meta-analysis by including the data from four studies which provided the risk of ischemic stroke in women with migraine with aura [11, 15, 17, 18]. We assumed no interaction between HC use and migraine with aura on the risk of ischemic stroke (*P* = 0.87) [17]. For migraine without aura we considered a pooled RR of ischemic stroke of 1.6 (95% CI 0.9–2.7, heterogeneity: Q = 8.05, df = 3, *p* = 0.045, I^2^ = 63%) using a random effect model meta-analysis by including the data from four studies which provided the risk of ischemic stroke in women with migraine without aura [11, 15, 17, 18]. We hypothesized no interaction between HCs use and migraine without aura in the risk of ischemic stroke (no data from available articles).

**Table 4:** Absolute risk of ischemic stroke in women aged 20 to 44 years in relation to the use of hormonal contraception and migraine status

	No migraine	Migraine with aura	Migraine without aura
Without hormonal contraception	2.5/100,000	5.9/100,000	4.0/100,000
With hormonal contraception	6.3/100,000	36.9/100,000	25.4/100,000

Data were calculated by using information provided in references #11,15,17,18,35

The absolute risk of ischemic stroke among young women who do not use HC is 2.5/100,000 per year whereas the same risk among young women who use HC is 6.3/100,000.

Considering women with migraine with aura, the risk of ischemic stroke in those young women who do not use HC is 5.9/100,000 per year whereas the same risk among those young women who use HC is 36.9/100,000 per year.

Considering women with migraine without aura, the risk of ischemic stroke in those young women who do not use HC is 4.0/100,000 per year whereas the same risk among those young women who use HC is 25.4/100,000 per year.

### Consensus statements

Statements are summarized in Table 5.

**Table 5:** Statements with the strength of the recommendation and the quality of evidence

Statement		Strength	Quality of evidence
1	In women who are seeking hormonal contraception, we recommend a clinical evaluation for the presence of migraine, for the definition of migraine subtype (i.e., with or without aura) and migraine frequency together with the ascertainment of conventional vascular risk factors before prescription of combined hormonal contraceptives	1, Strong	C, Low
2	In women who are seeking hormonal contraception, we recommend the use of a dedicated, easy-to-use tool to diagnose migraine and its subtypes (i.e., with and without aura)	1, Strong	C, Low
3	In women who are seeking hormonal contraception, we recommend consideration of the type of hormonal contraception taking into account their influence on the risk of ischemic stroke as there are high risk products (combined oral contraceptives containing >35 μg ethinylestradiol), medium risk products (combined oral hormonal contraceptives containing ≤35 μg ethinylestradiol, combined contraceptive patch, and combined vaginal ring) and no risk products (progestogen-only contraceptives including oral pill, subdermal implant, depot-injection, and levonorgestrel-releasing intrauterine system)	1, Strong	B, Medium
4	In women with migraine with aura who are seeking hormonal contraception, we suggest against prescription of combined hormonal contraceptives (oral pill, transdermal patch, and vaginal ring) containing ethinylestradiol and 17β-estradiol/estradiol valerate	2, Weak	C, Low
5	In women with migraine with aura who are seeking contraception we suggest non-hormonal contraception (condoms, copper-bearing intrauterine device, permanent methods) or progestogen-only contraceptives (oral pill, subdermal implant, depot-injection, and levonorgestrel-releasing intrauterine system) as the preferential option	1, Strong	C, Low
6	In women with migraine with aura who are already using combined hormonal contraceptives for contraception, we suggest switching to non-hormonal contraception (condoms, copper-bearing intrauterine device, permanent methods) or progestogen-only contraceptives (oral pill, subdermal implant, depot-injection, and levonorgestrel-releasing intrauterine system)	2, Weak	C, Low
7	In women with migraine without aura who are seeking hormonal contraception and who have additional risk factors (cigarette smoking, arterial hypertension, obesity, previous history of cardiovascular disease, previous history of deep vein thrombosis or pulmonary embolism), we suggest non-hormonal contraception (condoms, copper-bearing intrauterine device, permanent methods) or progestogen-only contraceptives (oral pill, subdermal implant, depot-injection, and levonorgestrel-releasing intrauterine system) as the preferential option	2, Weak	C, Low
8	In women with migraine without aura who are seeking hormonal contraceptives and who have no additional risk factors (cigarette smoking, arterial hypertension, obesity, previous history of cardiovascular disease, previous history of deep vein thrombosis or pulmonary embolism) we suggest the use of combined hormonal contraceptives containing ≤35 μg dose of ethinylestradiol as a possible contraceptive option with monitoring of migraine frequency and characteristics. Benefits and risk of combined hormonal contraceptives use in comparison to other contraceptive options have to be balanced carefully	2, Weak	C, Low
9	In women with migraine with aura or migraine without aura who require hormonal treatment for polycystic ovary syndrome or endometriosis we suggest to select the hormonal treatment of choice (progestogen-only or combined hormonal contraceptives) on clinical grounds	2, Weak	C, Low
10	In women who start combined hormonal contraceptives for contraception and who develop new onset of migraine with aura, or who develop new onset migraine without aura in a temporal relationship to starting the hormonal contraceptive, we suggest switching to non-hormonal contraception (condoms, copper-bearing intrauterine device, permanent methods) or progestogen-only contraceptives (oral pill, subdermal implant, depot-injection, and levonorgestrel-releasing intrauterine system).	2, Weak	C, Low
11	In women with migraine with or without aura who require emergency contraception, we suggest the use of levonorgestrel 1.5 mg orally, ulipristal acetate 30 mg orally, or the copper-bearing intrauterine device	2, Weak	C, Low
12	In women with migraine with or without aura seeking hormonal contraception, we suggest against specific tests (e.g. thrombophilia screening, patent foramen ovale evaluation or neuroimaging evaluation) to decide about hormonal contraceptive prescription unless those tests are indicated by the patient’s history or by the presence of specific symptoms	2, Weak	C, Low
13	In women with non-migraine headache who are seeking hormonal contraception any low-dose hormonal contraceptive can be used	2, Weak	C, Low

**Statement 1:** In women who are seeking hormonal contraception, we recommend a clinical evaluation for the presence of migraine, for the definition of migraine subtype (i.e., with or without aura) and migraine frequency together with the ascertainment of conventional vascular risk factors before prescription of combined hormonal contraceptives. 1C, Strong recommendation, Low quality of evidence.

Comment: Women who are seeking HC should be screened for the presence of vascular risk factors (i.e., arterial hypertension, cigarette smoking, obesity, previous history of cardiovascular disease, previous history of deep vein thrombosis or pulmonary embolism) which may increase the risk of cardiovascular events including ischemic stroke [40–42]. The Panel underscores the importance of evaluating those women also for the presence of migraine and of the definition of migraine subtypes (i.e., with or without aura). In fact, as detailed in the systematic review, available data indicated an increased risk of ischemic stroke in women with migraine using HC [1, 5, 11, 18, 26, 33, 34] and for this reason the presence of migraine deserves caution in prescription of HCs as detailed in those Statements. However, there are not enough data to address the risk of IS associated with the use of HC according to migraine subtypes (i.e., with or without aura) as most of the studies reported data for overall migraine only. Only two studies reported the risk of ischemic stroke in migraine with aura according to HC use [10, 17]. In the earlier study, the risk of ischemic stroke in migraineurs with aura was similar among HC users and nonusers, but the association among nonusers attained statistical significance owing to a larger sample size (OR, 1.5; 95% CI 1.1–2.1) [17]. In the more recent study using a comparative group of women without migraine and who were not using HCs as reference group, authors found that women with migraine with aura and active HC use had a 6-fold risk of ischemic stroke, while the risk was lower (OR 2.7; 95% CI, 1.9–3.7) in women with migraine with aura who were not using HCs [10]. In this same study authors reported evidence of an increased risk of ischemic stroke in women with migraine without aura either using (OR 1.8; 95% CI 1.1–2.9) or not using HC (OR 2.2; 95% CI 1.9–2.7) as compared to women without migraine and non-HC users. The study did not formally test whether the effect of migraine (with and without aura) on ischemic stroke was modified by HC intake status. However, the Panel underscores that definition of migraine subtype (i.e. with or without aura) is important to understand better the possible increase in the risk as several observational studies indicated that migraine with aura is associated with an increased risk of ischemic stroke [9–11, 13, 15, 17, 18, 43] while for migraine without aura, the interpretation of available data is more complex. In fact, some studies reported that migraine without aura is associated with an increased risk of ischemic stroke [10, 18, 19] whereas in others the association seemed present but values did not reach statistical significance [11, 15, 16].

The Panel suggests also to consider migraine frequency in women who are seeking hormonal contraception. There are not enough data to establish if also low migraine attack frequency (e.g. 4 attacks per year) is associated with the increased risk of ischemic stroke. However, migraine attack frequency for those with migraine with aura appears to be an issue for determining the risk of ischemic stroke. Findings from the *World Health Organization Collaborative Study of Cardiovascular Disease and Steroid Hormone Contraception* indicated that migraine with aura with migraine attacks more frequent than 12 times per year (OR 10.4; 95% CI 2.2–49.4) was associated with an increased risk of ischemic stroke [44]. Data from the *Stroke Prevention in Young Women Study* indicated that women with migraine with aura with a high migraine attack frequency (>12 attacks per year) had higher odds of stroke (OR 1.7; 95% CI 1.1–2.8), in addition to women with recent onset of migraine with aura (OR 8.3; 95% CI 2.6–25.7) [17]. According to data from the *Women’s Health Study*, the association between migraine with aura and ischemic stroke appeared J-shaped. Specifically, there were increased risks for a migraine attack frequency of less than monthly (HR 1.9; 95% CI 1.2–3.1) and greater or equal to weekly (hazard ratio [HR] 4.3; 95% CI 1.4–13.3), but not for monthly migraine attacks [45]. Additionally, in this same cohort there was evidence of the association between active (within the last year) migraine with aura and ischemic stroke whereas previous history of migraine was not associated with ischemic stroke risk [45].

**Statement 2:** In women who are seeking hormonal contraception, we recommend the use of a dedicated, easy-to-use tool to diagnose migraine and its subtypes (i.e., with and without aura). 1C, Strong recommendation, Low quality of evidence.

Comment: Migraine diagnosis is most reliable when established by a headache specialist using the ICHD criteria; however, a specialist diagnosis cannot be always obtained in women seeking HC; in that clinical setting, validated screening questionnaires may be useful to establish a headache diagnosis or to refer the appropriate patients to headache specialists. Ideally, a screening should initially assess whether women seeking HC suffer from recurrent headaches, then whether recurrent headaches are migraine and, finally, whether migraine is with or without aura. Types of migraine are not mutually exclusive and around 30% of people with migraine with aura also have attacks without aura [22, 46–48], with the pattern changing over time.

Several migraine screening tools have been tested, including two seminal questionnaires dating back to the 1990s [49, 50], the Migraine Screen Questionnaire (MS-Q) [51], the deCODE Migraine Questionnaire (DMQ3) [52], a German questionnaire [53], and the Structured Migraine Interview (SMI) [54]. ID-Migraine™ is a valid and reliable symptom-based screener for migraine without aura that has been developed for use in primary care [55]; the tool is available also in other languages than English, including Italian [56], Turkish [57], Portuguese [58], French [59], and Chinese [60]. It is based on the three best predictors for diagnosing migraine without aura, namely photophobia, disability and nausea; patients who report two of these symptoms have an 81% probability of having migraine and three symptoms increases the probability to 93% (Table 6).

**Table 6:** The Migraine-ID™ questionnaire

Identify your migraine		
Take the ID Migraine™ Quiz		
These 3 ID Migraine™ questions can help you learn more about your headaches or migraines.		
During the last 3 months did you have the following with your headaches:		
• You felt nauseated or sick to your stomach?	Yes	No
• Light bothered you (a lot more when you didn’t have headaches)?	Yes	No
• Your headaches limited your ability to work, study, or do what you needed to do for at least 1 day?	Yes	No
If you answered “yes”to 2 or more of the ID MigraineTM questions, you may suffer from migraines. It may help you tokeep a Migraine Diary so you can better talk to your doctor about your symptoms.		
If you answered “no” to these questions, you may not have migraine, but you should still discuss your symptoms with your doctor.		

From Lipton RB et al. Neurology 2003;61:375–382 [55]

A sensitive and specific tool is the visual aura rating scale (VARS) for migraine aura diagnosis, which is based on the ICHD diagnostic criteria [61]. The VARS score is the weighted sum of the presence of five visual symptom characteristics: duration 5–60 min (3 points), develops gradually over at least 5 min (2 points), scotoma (2 points), zig-zag lines (2 points) and unilateral (1 point) (Table 7). A VARS score of ≥5 out of a maximum score of 10 points has a sensitivity of 96% (95% CI 92–99%) and a specificity of 98% (95% CI 95–100%) for migraine aura.

**Table 7:** Visual Aura Rating Scale (VARS)

Visual symptom	Risk score
Duration 5–60 min	3
Develops gradually over 5 min	2
Scotoma	2
Zigzag line (fortification)	2
Unilateral (homonymous)	1
Migraine with aura diagnosis	≥5

From Eriksen MK et al. Cephalalgia 2005;25:801–810 [61]

A further available tool is represented by the LUMINA (Leiden University MIgraine Neuro-Analysis) web-based questionnaire [62]. The questionnaire was developed for the specific aim of being used in epidemiological studies. A seven-question subset of the questionnaire provided higher sensitivity (86% vs. 45%), slightly lower specificity (75% vs. 95%), and similar positive predictive value (86% vs. 88%) in assessing aura when comparing with the ICHD-II-based algorithm. The LUMINA web-based questionnaire allows the distinction between migraine with and without aura with a focus on visual aura symptoms [62]. However, currently the LUMINA has not been validated in clinical settings.

**Statement 3:** In women who are seeking hormonal contraception, we recommend consideration of the type of hormonal contraception taking into account their influence on the risk of ischemic stroke as there are high risk products (combined oral contraceptives containing >35 μg ethinylestradiol), medium risk products (combined oral hormonal contraceptives containing ≤35 μg ethinylestradiol, combined contraceptive patch, and combined vaginal ring) and no risk products (progestogen-only contraceptives including oral pill, subdermal implant, depot-injection, and levonorgestrel-releasing intrauterine system). 1B, Strong recommendation, Medium quality of evidence.

Comment: The risk of ischemic stroke associated with HC use mostly depends on the estrogen compound and is dose dependent [22, 63]. Initially, combined oral HCs contained estrogen in doses up to 150 μg of mestranol, a prodrug of ethinylestradiol. Over years, compounds containing lower estrogen doses were developed and marketed. The most common estrogen used is ethinylestradiol, currently in doses between 15 and 35 μg.

Combined oral HCs containing high dose of estrogen (**≥**50 μg) have been associated with an increased risk of ischemic stroke [23, 26, 64]. A meta-analysis reported a 4.5-fold increase in the relative risk of ischemic stroke (95% CI 2.2–9.5) in users of combined HCs containing ≥50 μg ethinylestradiol and of 2.8-fold (95% CI 2.0–3.9) in users of combined HCs containing 50 μg ethinylestradiol [23]. A more recent meta-analysis showed a 3.3-fold increase in the same risk in users of combined HCs containing ≥50 μg ethinylestradiol (95% CI 2.5–4.3) [26]. As HCs containing a lower estrogen dose are equally effective but safer (not only in terms of ischemic stroke risk) [23–26, 64, 65], those compounds containing high dose of estrogen are no longer the choice even for women without migraine. However, there are still available formulations containing 50 μg of estrogen available worldwide, but no more formulations with >50 μg of estrogen. Formulations that contain 50 μg estrogen account for less than 1% of contraceptives prescription in US [63].

Combined HCs containing lower estrogen doses are considered safer but nevertheless carry an increased risk of ischemic stroke in women in general. A meta-analysis reported a 2.1-fold increase in the relative risk of ischemic stroke (95% CI 1.6–2.8) in users of combined HCs containing <50 μg ethinylestradiol [23]. A further meta-analysis, limited to low-dose combined HCs, showed a 2.1-fold increase in users (95% CI 1.6–2.9) [24]. A more recent meta-analysis showed a 1.8-fold increase in the relative risk of ischemic stroke (95% CI, 1.6–1.9) in users of combined HCs containing 30–40 μg ethinylestradiol and of 1.6-fold (95% CI 1.4–1.8) in users of combined HCs contraceptives containing 20 μg ethinylestradiol [26]. A recent observational study, on a large cohort of French women, indicated that combined HCs with 20 μg ethinylestradiol were associated with a reduced relative risk of ischemic stroke as compared to pills containing 30–40 μg ethinylestradiol (adjusted RR 0.8; 95% CI 0.7–1.0) [65].

The combined HCs transdermal patch and vaginal ring are relatively new contraceptive methods. They act by releasing hormones into systemic circulation. An open-label, randomized study showed that exposure to ethinylestradiol is lower in those who use the ring as compared to those who use the patch or the pill [66]. Additionally, the ring provides lower variation in serum ethinylestradiol levels as compared to the patch or to the ring [66]. Because the vaginal ring and transdermal patch routes of administration avoid hepatic first-pass metabolism, the impact on hepatic induction of coagulation factors might be different from users of oral formulations [67]. Safety may depend on individual absorption levels of ethinylestradiol but according to available evidence, the non-oral formulations provide a comparable safety and pharmacokinetic profile to combined oral HCs with similar hormone formulations [42]. Limited and conflicting information is available on the safety of these methods regarding vascular events [68–76]. Two studies examined the association between the combined HC transdermal patch use and arterial thromboembolism but were unable to demonstrate an increased risk; however, those studies could have been underpowered [68, 72]. A further study found a non-significant 3.2-fold increase in the relative risk of ischemic stroke among users of the combined HC transdermal patch (95% CI 0.8–12.6) and a 2.5-fold increase in the same risk among users of the combined HC vaginal ring (95% CI 1.4–4.4) [74]. A further cohort study did not find an increased risk of arterial thrombotic events (including ischemic stroke) in women who initiated treatment with combined HC transdermal patch or combined HC vaginal ring as compared to use of low-dose (10–35 μg) ethinylestradiol combined contraceptive [75]. A recent systematic review pointed out that evidence did not demonstrate an increased risk of arterial thromboembolism among women using the combined HC transdermal patch [76]. Additional studies are needed to further clarify any risk among users of non-oral combined HCs. Of note no specific data are available regarding the safety of those compounds in women with migraine.

Several different progestogens are available in current combined HC formulations. Progestogens are classified into first (norethisterone), second (norgestrel, levonogestrel), third (desogestrel, gestodene norgestimate), and fourth (drospirenone) generation compounds. New generation progestogens were developed to reduce side effects having less androgenic properties. The newer progestogens also enabled the use of low-dose estrogen formulations. Although they have better lipid profiles and promote less insulin resistance compared to 2nd generation progestogens, the 3rd generation progestogens failed to reduce the risk of stroke and myocardial infarction. There is no substantial difference in the ischemic stroke risk among the different progestogens contained in combined HCs [67, 77]. A meta-analysis showed, for <50 μg ethinylestradiol pills, the relative risk associated with first-, second-, and third generation progestogens was 2.2 (95% CI 1.1–4.3), 2.9 (95% CI 2.2–3.8), and 2.5 (95% CI 0.8–6.2), respectively [23]. In a further meta-analysis, the relative risk of ischemic stroke associated with pills containing second- and third-generation progestogens was 2.5 (95% CI 2.0–3.3) and 2.0 (95% CI 1.2–3.6), respectively [24]. A further study showed that the risk of ischemic stroke did not differ significantly according to the type of progestogen in users of combined HCs containing 30–40 μg ethinylestradiol [74]. Third and 4th generations progestogens, when used in combined formulations, may also be associated with an increased risk of venous thromboembolism [78, 79]. Development of arterial thrombosis is most likely due to estrogen effects of combined HCs on the coagulation system. Available data indicated that there is no increased risk of ischemic stroke associated with progestogen-only [18, 20, 80–86], including progestogen-only injectable (primarily medroxyprogesterone acetate) [86], subdermal implants [74, 84], the levonorgestrel intrauterine system [74] and progestogen-only pills [18, 20, 74, 81, 86]. A meta-analysis of 6 case-control studies of progestogen-only HCs showed a pooled risk of 1.0 (95% CI 0.7–1.3) [80]. These data were further supported by a recent pooled analysis of data which indicated that progestogen-only HCs were not associated with an increased risk of ischemic stroke (OR, 1.0; 95% CI, 0.7–1.4) [26].

**Statement 4:** In women with migraine with aura who are seeking hormonal contraception, we suggest against prescription of combined hormonal contraceptives (oral pill, transdermal patch, and vaginal ring) containing ethinylestradiol and 17β-estradiol/estradiol valerate. 2C, Weak recommendation, Low quality of evidence.

Comment: As reported earlier in this text, combined HCs containing low dose of ethinylestradiol, even if safer than compounds containing higher dose, have also been associated with an increased risk of ischemic stroke [23, 24, 26]. The relative increase in the risk of ischemic stroke with the use of combined oral formulations containing an ethinylestradiol dose between 20 and 40 μg is about 2-fold [25, 74]. However, the absolute risk of ischemic stroke is small due to the low incidence of the disease in healthy young women [37]. Although ischemic stroke events are overall rare among women of reproductive age they can have devastating complications associated with significant morbidity and mortality. Despite the overall low absolute risk of ischemic stroke from combined HCs, certain subgroups of women, including those with migraine with aura, may be at higher risk of stroke. In fact, as reported in the systematic review, some studies indicated that use of HCs in women with migraine is associated with further increase in the risk of ischemic stroke [17, 34]. As migraine with aura is a risk factor for ischemic stroke [8–18] use of combined HCs is contraindicated in women with this condition as supported also by the World Health Organization [42], the UK Faculty of Sexual and Reproductive Healthcare [41] and the US Centers for Disease Control and Prevention [40]. Women are not denied effective contraception as other methods are available. However, the Panel points out that further studies should address the possible threat driven by the association between HCs use in women with migraine. In fact, most of the studies which indicated an increased risk of ischemic stroke in women with migraine lacked some specific data. First of all, in most of those studies there was no information according to migraine subtype (i.e. with or without aura). However, this may represent a limitation mostly for migraine without aura, which (as detailed later in this text) has not been reliably associated with an increased risk of ischemic stroke. For migraine with aura, available studies are more homogeneous in indicating an association with increased risk of ischemic stroke. The other point of lack of evidence refers to the dose of ethinylestradiol. Even though estrogen dose is related to the risk of stroke in the general female population, as described in the comment to Statement 3, it remains unclear how the estrogen dose could impact on the risk of ischemic stroke in women with migraine [1, 5, 11, 17, 18, 26, 33, 34]. Only two studies provided the risk of ischemic stroke in women with migraine according to ethinylestradiol dose [11]. In the first study, women using <50 μg ethinylestradiol dose were included [34]. In this study, the relative risk of ischemic stroke was increased by 2.1-fold in current HCs users (95% CI, 1.2–3.7) who had migraine but the same risk was not elevated among women without such a history as compared to HCs non-users [34]. In the second study the relative risk of ischemic stroke was increased by 16.9-fold in migraineurs who were HC users (95% CI 2.7–106) but the study was unable to demonstrate an association in migraineurs who were HC non-users as compared to non-migraineurs and non-users [11]. When analysis was stratified by estrogen dose authors found a non-significant increase in the relative risk of ischemic stroke in migraineurs who were users of low (<50 μg) estrogen dose (OR 6.6; 95% CI 0.8–54.8) whereas the risk for higher (≥50 μg) doses could not be computed in that study [11].

Combined HCs containing estradiol, an endogenous ovarian hormone, have been developed [67, 78] to reduce risk of thrombotic events associated with ethinylestradiol. Pills containing micronized 17β-estradiol and estradiol valerate are currently available on the market. Pills containing estradiol in similar levels to the natural hormone cycle should be in theory associated with a relatively lower risk of ischemic stroke compared to the synthetic ethinylestradiol. A preliminary study suggested that the risk of venous thromboembolism with the estradiol valerate/dienogest was lower than with 3rd and 4th generation combined HCs and higher than a levonorgestrel/ethinylestradiol pill [Lidegaard O, personal communication]. In the same report, the risks for acute myocardial infarction and thrombotic stroke appeared to be lower with dienogest/estradiol valerate than with 2nd generation combined HCs. Some preliminary data indicate that combined HCs containing estradiol valerate are associated with lower cardiovascular risk as compared to combined HCs containing ethinylestradiol [87]. The *International Active Surveillance study Safety of Contraceptives: Role of Estrogens* (INAS-SCORE) was an observational study investigating the cardiovascular risks associated with the use of a combined HCs containing dienogest and estradiol valerate compared to established combined HCs (mostly containing ethinylestradiol and levonorgestrel) in a routine clinical setting in the United States and Europe [87]. The study indicated that the dienogest and estradiol valerate pill is associated with similar or even lower cardiovascular risk compared to levonorgestrel containing combined HCs and other combined HCs [87]. However, as the study follow-up was relatively short (mean 2 years) and number of events was low no firm conclusions could be drawn. Additionally, no information was available on migraine status. At the moment, there is not enough evidence to conclude about the cardiovascular safety of combined HCs containing estrogens other than ethinylestradiol. Until such evidence will become available, combined pills with natural estrogen should be considered as other types of combined pills.

**Statement 5:** In women with migraine with aura who are seeking contraception we suggest non-hormonal contraception (condoms, copper-bearing intrauterine device, permanent methods) or progestogen-only contraceptives (oral pill, subdermal implant, depot-injection, and levonorgestrel-releasing intrauterine system) as the preferential option. 1C, Strong recommendation, Low quality of evidence.

Comment: Progestogen-only contraceptives include progestogen-only pills, subdermal implants, and intrauterine systems. Progestogen-only contraceptives are associated with more breakthrough bleeding and, in some formulations, lower contraceptive efficacy than combined HCs [78]. Moreover, depot preparations of medroxyprogesterone acetate have been linked to reversible decreases in bone density [78]. Though there is a debate [83, 88] about whether different progestogens impact the risk of venous thromboembolism, progestogens do not appear to affect the risk of arterial events [64]. In fact, the cardiovascular risk associated with combined HCs, has been mainly attributed to the estrogen component which exerts a strong effect on the coagulation system. In two meta-analyses, progestogen-only contraceptives have not been associated with an increased risk of ischemic stroke [26, 80]. There are no studies that specifically tested the safety of those compounds in women with migraine regarding ischemic stroke risk. Only one study clearly indicated that subjects using progestogen-only were included, but those compounds were used by less than 5% of all women with migraine and no results were reported according to hormonal contraceptive type [18]. In the absence of clear evidence on the risk of ischemic stroke associated with the use of progestogen-only contraceptives in women with migraine, currently indirect evidence does not link the use of those compounds with an increased risk of arterial events including ischemic stroke (for additional information refer to comment to Statement 3) [76]. For those reasons, there are no issues which may contraindicate their use in subjects with migraine. Additionally, some studies indicated that the use of progestogen-only contraceptives in women with migraine is associated with significant reduction in migraine attack frequency, migraine intensity, use of triptans and pain score and in improvement in quality of life [89–95].

**Statement 6:** In women with migraine with aura who are already using combined hormonal contraceptives for contraception, we suggest switching to non-hormonal contraception (condoms, copper-bearing intrauterine device, permanent methods) or progestogen-only contraceptives (oral pill, subdermal implant, depot-injection, and levonorgestrel-releasing intrauterine system). 2C, Weak recommendation, Low quality of evidence.

Comment: No studies provided reliable clinical information to establish whether the risk of having ischemic stroke associated with the use of combined HCs in women with migraine declines with long-time use. However, a clear close temporal relationship between initiation of combined HCs and ischemic stroke onset has not been identified. For this reason, use of those compounds should be discontinued whenever the risk factor migraine with aura is recognized. This suggestion is even more stringent in those subjects who experience high migraine attack frequency. In fact, as reported earlier in this text some preliminary data indicate that migraine attack frequency of women with migraine with aura appears to be an issue for determining the risk of ischemic stroke [17, 44, 45] as the increased risk seems to be carried by high migraine attack frequency rather than sporadic attacks.

**Statement 7:** In women with migraine without aura who are seeking hormonal contraception and who have additional risk factors (cigarette smoking, arterial hypertension, obesity, previous history of cardiovascular disease, previous history of deep vein thrombosis or pulmonary embolism), we suggest non-hormonal contraception (condoms, copper-bearing intrauterine device, permanent methods) or progestogen-only contraceptives (oral pill, subdermal implant, depot-injection, and levonorgestrel-releasing intrauterine system) as the preferential option. 2C, Weak recommendation, Low quality of evidence.

Comment: For migraine without aura, the interpretation of available data is rather complex as some studies reported that migraine without aura is associated with an increased risk of ischemic stroke [10, 18, 19] whereas in others the association was not confirmed [11, 15, 16]. Further, the definition of a clear association between migraine without aura and ischemic stroke is even more complex as some studies linking migraine with ischemic stroke risk had no information on migraine aura [2, 18, 20] and because the challenge of migraine aura diagnosis in population-based studies. It should also be considered that data, which showed the increased risk of ischemic stroke in women with migraine, mostly refer to migraine overall and that migraine without aura accounts for most migraines. Considering those issues, probably migraine without aura carries some risk of ischemic stroke even if this risk is lower than that observed in subjects with migraine with aura. Additionally, one recent study indicated an increased risk of ischemic stroke in women with migraine without aura using HCs (OR 1.8; 95% CI 1.1–2.9) [10]. The risk of ischemic stroke in this study was also increased in women with migraine without aura not using HCs (OR 2.2, 95% CI 1.9–2.7). However, in this study authors did not provide the risk of ischemic stroke in migraineurs without aura using HCs versus not using HCs. Unless new studies will provide more clear evidence about the risk of ischemic stroke in women with migraine without aura using HCs, the Panel suggest to privilege safety and methods which do not carry any increased risk of ischemic stroke in women with migraine without aura with additional risk factors. This position is in line with the content of the medical eligibility criteria by the World Health Organization [42], the UK Faculty of Sexual and Reproductive Healthcare [41]and the US Centers for Disease Control and Prevention [40].

**Statement 8:** In women with migraine without aura who are seeking hormonal contraceptives and who have no additional risk factors (cigarette smoking, arterial hypertension, obesity, previous history of cardiovascular disease, previous history of deep vein thrombosis or pulmonary embolism) we suggest the use of combined hormonal contraceptives containing ≤35 μg dose of ethinylestradiol as a possible contraceptive option with monitoring of migraine frequency and characteristics. Benefits and risk of combined hormonal contraceptives use in comparison to other contraceptive options have to be balanced carefully. 2C, Weak recommendation, Low quality of evidence.

Comment: As combined HCs may have also non-contraceptive benefits the Panel supports their possible use in women with migraine without aura in the absence of any other factor which could potentially increase the risk of ischemic stroke. Non-contraceptive benefits of combined HCs include prevention of cancer [96, 97]. Additionally, combined HCs may have different impact on the course of migraine and in some cases improvements may be appreciated [98, 99]. As data indicated that migraineurs may have increased burden of some cardiovascular risk factors as compared to non-migraineurs, careful screening is needed [100]. In case of use, monitoring of migraine characteristics may be relevant and cessation of the compound in the presence of worsening of frequency or severity. As data indicated that active rather than past migraine is associated with increased risk of ischemic stroke as also high migraine attack frequency [15] we suggest careful review and possibly change prescription if migraine changes towards a worsening pattern after initiation of combined HCs.

**Statement 9:** In women with migraine with aura or migraine without aura who require hormonal treatment for polycystic ovary syndrome or endometriosis we suggest to select the hormonal treatment of choice (progestogen-only or combined hormonal contraceptives) on clinical grounds. 2C, Weak recommendation, Low quality of evidence.

Comment: In the presence of a medical condition requiring hormonal treatment there is a different risk/benefit profile. Polycystic ovary syndrome (PCOS) is a common gynecological disorder associated with hyperandrogenism and menstrual disorders with chronic anovulation, infertility hirsutism, acne and obesity [101]. Endometriosis is characterized by the presence of endometrial-like tissue outside the uterus and is associated with a chronic inflammatory reaction; its main symptoms are pain and infertility. In PCOS additionally, patients often suffer from metabolic disorders: insulin resistance, hyperinsulinemia, dyslipidemia, leading to atherosclerosis and other irregularities of the metabolic syndrome. Chronic inflammation usually accompanies also PCOS. Additionally, these patients often suffer from metabolic disorders: insulin resistance, hyperinsulinemia, dyslipidemia, leading to atherosclerosis and other irregularities of the metabolic syndrome. Because of the metabolic abnormalities observed in patients with PCOS, they are in the high-risk group for development of cardiovascular diseases [102–104]. Women with PCOS should have medical care from the time of diagnosis. It should consist not only in the treatment of hormonal disorders and infertility, but also in early diagnosis, prevention and treatment of metabolic disorders. This will reduce the risk of cardiovascular disease and its complications in the future and improve the patient’s quality of life. Additionally, some data suggest that HCs may have a favorable effect on the risk of vascular diseases in women with PCOS [105].

**Statement 10:** In women who start combined hormonal contraceptives for contraception and who develop new onset of migraine with aura, or who develop new onset migraine without aura in a temporal relationship to starting the hormonal contraceptive, we suggest switching to non-hormonal contraception (condoms, copper-bearing intrauterine device, permanent methods) or progestogen-only contraceptives (oral pill, subdermal implant, depot-injection, and levonorgestrel-releasing intrauterine system). 2C, Weak recommendation, Low quality of evidence.

It is well known that combined HCs may impact on the course of migraine [106]; the impact may consist of worsening of previous migraine, in developing de novo migraine (with or without aura), or in some cases in improving migraine. Some women do not experience any headache change associated with the use of combined HCs. Some women do appear to have a higher risk of headache exacerbation or new-onset headache attributable to combined HC use. This mostly occurs with the use of combined HCs that provide a drop estrogen that is equivalent to the end-luteal phase drop. This higher risk is most apparent in women with a strong personal or family history of troublesome headaches, particularly migraine [67]. The risk also increases with age. Even within the higher risk groups, some women note improvement in headache with combined HCs use. In several women reporting initial worsening, headache complaints decrease with continued use. It is not always easy and obvious to establish a clear relationship between migraine onset or worsening and use of combined HCs. In fact, migraine typically starts in teens/twenties, so association with HCs use may be coincidental. It is worth to consider that any change in migraine pattern is only likely to be associated with hormone use if there has been a clear temporal relationship. An increase in migraine frequency several years after starting HCs is more probably associated with independent, non-hormonal triggers. Headache that is related to combined HCs use generally is precipitated by estrogen withdrawal during the pill-free or placebo pill week of treatment and causal relationship is probably more definite when attacks occur regularly during hormone-free interval. Continuous treatment may ameliorate attacks occurring in the pill-free or placebo pill interval of treatment.

There are no studies which have addressed whether changing from migraine without aura into migraine with aura, associated with initiation of combined HC, is associated with an increased risk of vascular events including ischemic stroke. However, there are some old data, from studies of high-dose combined HCs, which suggest that the development of migraine aura in women using those compounds correlates with increased platelet activation [107, 108]. The *Womens’ Health Study* showed that only active migraine with aura was associated with increased risk of ischemic stroke (OR 1.9, 95% CI: 1.2–3.1) whereas prior migraine, more than 1 year before entry into the study (OR 0.8; 95% CI 0.4–1.4), was not associated with increased risk of any ischemic event at follow up. However, there are no data regarding how long a women already had their migraine prior to study entry and how changes of migraine status during the 11.9 year follow-up affect the results [15]. Additionally, we have also to consider that there are no data available whether improvement of migraine with HCs use, as well with any other preventative treatment, are associated with decrease the risk of vascular events.

**Statement 11:** In women with migraine with or without aura who require emergency contraception, we suggest the use of levonorgestrel 1.5 mg orally, ulipristal acetate 30 mg orally, or the copper-bearing intrauterine device. 2C, Weak recommendation, Low quality of evidence.

Comment: Emergency contraception, or post-coital contraception, refers to methods of contraception that can be used to prevent pregnancy after sexual intercourse. There are 2 methods of hormonal emergency contraception: progestin-only pills (levonorgestrel) and progesterone receptor modulator pill (ulipristal acetate); the copper-bearing intrauterine device can be also used as non-hormonal method. There are no reliable data that systematically addressed the risk of ischemic stroke associated with emergency contraception. However, as the duration of use of emergency contraceptive pills is less than the duration of regular use of combined HCs and they would be expected to have less clinical impact on ischemic stroke risk in women with migraine. Some case reports link emergency contraception to stroke occurrence [109–113]. However, those data do not allow to reliably establish a causal relationship between emergency contraceptive use and ischemic stroke due to the lack of a control group. In two [109, 110] of those reports the emergency contraceptive was represented by two tablets of levonorgestrel 250 mg plus ethinylestradiol 50 mg 19 h before presentation and a second dose of two tablets 7 h before presentation (e.g. a total of four tablets and 200 mg of ethinylestradiol in a 12-h period of time). This method has currently been superseded. In a further report the ischemic stroke was associated with the use of levonogerstrel 1 mg plus ethinylestradiol 0.20 mg [113]. In a fourth case report the emergency contraceptive pill was represented by levonorgestrel 1.5 mg but the pill had been taken only once, 3 months prior to stroke onset, and so the causal relationship appears weak [111]. The same pill was associated with ischemic stroke occurrence in a further report, but in this case the pill had been taken the day before stroke onset making more possible a causal relationship [112].

**Statement 12:** In women with migraine with or without aura seeking hormonal contraception, we suggest against specific tests (e.g. thrombophilia screening, patent foramen ovale evaluation or neuroimaging evaluation) to decide about hormonal contraceptive prescription unless those tests are indicated by the patient’s history or by the presence of specific symptoms. 2C, Weak recommendation, Low quality of evidence.

Comment: Little is known about a possible risk profile predisposing women with migraine to ischemic stroke. Despite for most women with migraine combined HCs are safe and highly effective methods of contraception with added non-contraceptive health benefits some women with migraine may experience an ischemic stroke associated with the use of HCs. There are no reliable markers which may be used to select those women with migraine in whom HC use may lead to ischemic stroke. Prothrombotic factors may potentially increase the risk of ischemic stroke associated with combined HC use [114–116]. However, thrombophilia is a very rare condition and available tools screens only identify currently known factors but others may exist for which there is no screening yet. Most women with ischemic stroke, associated or not with migraine and use of HC, do not have recognized hereditary coagulation problems. A systematic review and meta-analysis addressed the possible benefits of thrombophilia screening for venous thromboembolism risk in the setting of combined HC use [117]. Authors found that despite combined HC use was associated with an increased risk of venous thromboembolism in patients with thrombophilia, the benefits of screening were modest because of the low absolute risk due to the low prevalence of thrombophilias. As ischemic stroke is much less common than venous thromboembolism [74] the yield of routine screening would be even lower for ischemic stroke. Any possible test would involve costs which are not sustainable unless benefits of the screening test have been proven. Data about the association between migraine and patent foramen ovale are controversial. Several studies showed an increased prevalence of patent foramen ovale in subjects with migraine as compared to non-migraineurs [118–120]. The only population-based study investigating this association found no relationship between those two conditions but this study included mostly subjects of non-reproductive age [121]; this was not different when only considering migraine with aura. A meta-analysis including case-control studies demonstrated a 2.5-fold increased (95% CI 2.0–3.1) prevalence of patent foramen ovale in patients with migraine and a 5.1-fold (95% CI 4.7–5.6) increased prevalence of migraine in patients with patent foramen ovale [119]. The relationship between migraine with aura, ischemic stroke, and patent foramen ovale remains not entirely clear and it is possible that it may be relevant in a subset of patients [122]. However, in the majority of patients with migraine there is no clear involvement of patent foramen ovale in the increased risk of ischemic stroke. Several studies have also indicated that compared to individuals without migraine, patients with migraine have a higher burden of asymptomatic white matter brain lesions and, according to some studies, infarct-like lesions on brain magnetic resonance imaging [123–126]. Those lesions may suggest chronic ischemic disease but their nature remains elusive because of lack of neuropathological correlation. However, there is insufficient evidence to suggest that those alterations represent markers of increased stroke risk in patients with migraine.

**Statement 13:** In women with non-migraine headache who are seeking hormonal contraception any low-dose hormonal contraceptive can be used. 2C, Weak recommendation, Low quality of evidence.

Comment: Few studies evaluated the risk of ischemic stroke in subjects with headache other than migraine [14, 127–131]. Currently, there is no evidence that reliably indicates that non-migraine headaches are associated with an increased risk of ischemic stroke. The association remains unknown in young women as the available studies mostly involved older subjects. One study, involving subjects aged ≥60 years, did not find an association between non-migraine headache and ischemic stroke; however, the number of included subjects in this study was low and it may have been underpowered to demonstrate a significant association [129]. A second study showed an increased risk of total stroke after 1 year of follow-up in men with headache (HR 3.9; 95% CI 2.0–7.8), which leveled off over the course of the remaining follow-up but remained increased [127]. In women, however, such an association could not be established [127]. A further study found increased risk of stroke among men and women who reported analgesic use for headache in general, but the classification of headache and stroke in this study was imprecise [128]. In addition, data from the *Women’s Health Study* did not show an increased risk of ischemic stroke in women aged ≥45 years with headache in general or non-migraine headache [14]. A recent study in Asians, including subjects aged ≥18 years, found that tension type headache was associated with increased risk of ischemic stroke (HR 2.3; 95% CI 1.2–2.8) [131]. However, as diagnosis of tension type headache was based on using administrative coding data only, further studies using validated diagnoses are needed to establish a possible association between the two conditions. Another recent study involving subjects aged >65 years indicated that whereas subjects with migraine had no increased risk of any stroke, subjects with non-migraine headache were twice as likely to have any stroke (HR 2.0; 95% CI 1.0–3.9) [130].

## Discussion

Evidence addressing the risk of ischemic stroke associated with the use of HCs is generally poor. All information relies on observational data [23–26, 64, 65, 68–76, 80–88, 132], which may carry the risk of potential bias. Available studies had different settings and used different groups for comparing risks, limiting reliable comparison of studies as a pooled analysis of data. Most of the available studies were published several years ago and used compounds which are different from those available today. Additionally, in most studies not enough information is available regarding the type of HCs considered and in most cases results are not provided according to migraine type. Consequently, much efforts are needed to further investigate the possible risk associated with the use of HCs in women with migraine. Despite those limitations, available data pointed toward an increased risk of ischemic stroke associated with the use of HCs in women with migraine. Considering this evidence, and unless studies will prove safety of the use of combined HCs in women with migraine, the present recommendations from this Consensus Group give priority to safety and suggest several limitations in the use of combined HCs in women with migraine. However, according to available evidence it cannot be excluded that currently available combined HCs are safer than those included in the studies reviewed, particularly the older studies, and that future recommendations may be less restrictive. But at present, we believe that caution is mandatory. In fact, even if the absolute risk associated with the use of combined HC may not be high, the consequences of an ischemic stroke may be devastating for patients and their families. There are alternative methods which provide similar contraceptive benefits but that are much safer in terms of risks. The present recommendations support the use of those methods as preferential contraceptive option in women with migraine. As combined HCs exert some non-contraceptives benefits such as a protecting role against endometrial, ovarian, and bowel cancer, future studies should consider combined endpoints to globally address benefits and risk related to the use of combined HCs. Previous Recommendations about use of HC in women with migraine were published by the International Headache Society in 2000 [133]. In that document, the Authors did not contraindicate combined HC use in the absence of migraine with aura or additional risk factors for ischemic stroke. The present document provides more details referring to type of HC as to different situations and comorbidities even in women with migraine without aura.

The Consensus Group considers that it is necessary to conduct further research to identify safe HC methods for women with migraine (i.e. evaluation of the different doses of ethinylestradiol, of the risk associated with natural estrogens, and the risk associated with the different progestogen formulations), to clarify mechanisms linking HCs to increased stroke risk in women with migraine, and to identify subgroups of migraineurs with high risk of stroke. Future studies should assess the risk of ischemic stroke in women with migraine according to migraine subtype (i.e. with or without aura) using combined HCs (including dose and type of hormones) or progestogen-only contraceptives versus those women with migraine not using combined HCs or progestogen-only contraceptives. Another point of further research is to clarify the risk of ischemic stroke associated with combined HCs containing natural estrogens versus ethinylestradiol. Further data are also needed to better define the risk and benefits of non-oral combined HCs (combined contraceptive transdermal patch and combined vaginal ring). Additionally, basic research studies should try to understand why combined HCs increase the risk of ischemic stroke and the specific mechanisms leading to the vascular events in women with migraine. Studies should try to identify possible markers for the increased risk of stroke in migraineurs and for the risk of developing thrombosis associated with the use of HCs. Future studies should also try to understand how migraine features (e.g., frequency or duration of the disease) may impact on the risk of ischemic stroke associated with HC use and if there are age groups at particularly high risk.

### Statement supporting group

Lukas Hefler, MD, Ordensklinikum Linz, Austria.

Katie Linstra, MD; Erasmus Medical Centre, Rotterdam, and Leiden University, Medical Centre, Leiden, The Netherlands.

Silvia Martella, MD, University of Pavia, Pavia Italy.

Raffaele Ornello, MD; University of L’Aquila, L’Aquila, Italy.

Francesca Pistoia, MD, PhD; University of L’Aquila, L’Aquila, Italy.
