# Correction to: Hormonal contraceptives and risk of ischemic stroke in women with migraine: a consensus statement from the European Headache Federation (EHF) and the European Society of Contraception and Reproductive Health (ESC)

**DOI:** 10.1186/s10194-018-0912-9

**Published:** 2018-09-10

**Authors:** Simona Sacco, Gabriele S. Merki-Feld, Karen Lehrmann Ægidius, Johannes Bitzer, Marianne Canonico, Tobias Kurth, Christian Lampl, Øjvind Lidegaard, E. Anne MacGregor, Antoinette MaassenVanDenBrink, Dimos-Dimitrios Mitsikostas, Rossella Elena Nappi, George Ntaios, Per Morten Sandset, Paolo Martelletti, on behalf of the European Headache Federation (EHF) and the European Society of Contraception and Reproductive Health (ESC)

**Affiliations:** 1 Department of Applied Clinical Sciences and Biotechnology, University of L’Aquila, L’Aquila, Italy; 2 Department of Gynecology, Clinic for Reproductive Endocrinology, University Hospital, Zürich, Switzerland; 3 Department of Neurology, Bispebjerg Hospital and University of Copenhagen, Copenhagen, Denmark; 4 Department of Obstetrics and Gynecology, University Hospital of Basel, Basel, Switzerland; 5 Université Paris-Saclay, University Paris-Sud, UVSQ, CESP, Inserm UMRS1018, Orsay, France; 6 Institute of Public Health, Charité – Universitätsmedizin Berlin, Berlin, Germany; 7 Headache Medical Center Seilerstaette Linz, Linz, Austria; 8 Department of Geriatric Medicine Ordensklinikum Linz, Linz, Austria; 9 Department of Obstetrics & Gynaecology, Rigshospitalet, Faculty of Health Sciences, University of Copenhagen, Copenhagen, Denmark; 10 Centre for Neuroscience & Trauma, BICMS, Barts and the London School of Medicine and Dentistry, London, UK; 11 Barts Sexual Health Centre, St Bartholomew’s Hospital, London, UK; 12 Division of Vascular Medicine and Pharmacology, Department of Internal Medicine, Erasmus Medical Center Rotterdam, Rotterdam, The Netherlands; 13 Department of Neurology, Aeginition Hospital, National and Kapodistrian University of Athens, Athens, Greece; 14 Research Centre for Reproductive Medicine, Gynecological Endocrinology and Menopause, IRCCS S. Matteo Foundation, Department of Clinical, Surgical, Diagnostic and Pediatric Sciences, University of Pavia, Pavia, Italy; 15 University Consortium for Adaptive Disorders and Head Pain (UCADH), University of Pavia, Pavia, Italy; 16 Department of Medicine, University of Thessaly, Larissa, Greece; 17 Department of Haematology, Oslo University Hospital and University of Oslo, Oslo, Norway; 18 Department of Clinical and Molecular Medicine, Sapienza University of Rome, Rome, Italy; 19 Regional Referral Headache Centre, Sant’Andrea Hospital, Rome, Italy

## Correction

Following the publication of this article [1], the authors noticed that they incorrectly reported the Absolute risk of ischemic stroke in women aged 20 to 44 years in relation to the use of hormonal contraception and migraine status due to a miscalculation. They apologize for this misreported result.

The correct version of Table 4 has been included in this correction.

**Table 4:** (Revised) Absolute risk of ischemic stroke in women aged 20 to 44 years in relation to the use of hormonal contraception and migraine status

	No migraine	Migraine with aura	Migraine without aura
Without hormonal contraception	2.5/100,000	5.9/100,000	4.0/100,000
With hormonal contraception	6.3/100,000	14.5/100,000	10.0/100,000

Considering women with migraine with aura, the risk of ischemic stroke in those young women who do not use HC is 5.9/100,000 per year whereas the same risk among those young women who use HC is 14.5 /100,000 per year

Considering women with migraine without aura, the risk of ischemic stroke in those young women who do not use HC is 4.0/100,000 per year whereas the same risk among those young women who use HC is 10.0/100,000 per year
